# Aflatoxin contamination of animal feeds and its predictors among dairy farms in Northwest Ethiopia: One Health approach implications

**DOI:** 10.3389/fvets.2023.1123573

**Published:** 2023-03-23

**Authors:** Fitalew Tadele, Biruk Demissie, Alebachew Amsalu, Habtamu Demelash, Zelalem Mengist, Argaw Ambelu, Chalachew Yenew

**Affiliations:** ^1^Department of Biomedical Sciences, College of Health Sciences, Debre Tabor University, Gondar, Ethiopia; ^2^Department of Public Health, College of Health Sciences, Debre Tabor University, Gondar, Ethiopia; ^3^Department of Animal Health, College of Veterinary Medicine, Mekelle University, Mek'ele, Ethiopia; ^4^Division of Water and Health, Ethiopian Institute of Water Resources, Addis Ababa University, Addis Ababa, Ethiopia

**Keywords:** zoonotic pathogens, contamination, One Health approach, Ethiopia, environmental factor

## Abstract

**Background:**

In dairy farming, animal feed is the first line of food safety. Animal feed can become contaminated and spoiled on farms or in storage facilities due to the diverse microorganisms that are naturally present around or on various animal feeds. This study aims to assess the level of aflatoxin and predictors in animal feeds among dairy farms in the South Gondar Zone of Ethiopia.

**Methods:**

A total of 100 samples of each animal feed ingredient (atella, hay, commercial concentrates, and cut and carry pasture) were obtained. A total of 400 animal feed ingredient samples were tested separately among 100 randomly chosen dairy farmers for aflatoxin analysis. Simultaneously, swabs from cow udders and water samples were also collected. Using a structured and pretested questionnaire, the knowledge and practices of animal feed administrators responsible for managing animal feed were also evaluated. Descriptive statistics and logistic regression models were used to identify determinants.

**Results:**

From the total animal feed analyzed, 96% was positive for aflatoxins. Feed storage facilities, feed storage duration, education of animal feed administrators, mixed concentrates, and previous training were found to be associated with aflatoxin contamination levels in animal feeds.

**Conclusions:**

The levels of aflatoxin contamination in animal feeds were found to be higher than the recommended limit; these findings suggest the spread of aflatoxin contamination between humans and animals. Furthermore, the occurrence of aflatoxins in the environment results from milk becoming contaminated with aflatoxins. A One Health strategy should therefore receive special consideration to tackle such problems and safeguard consumer safety.

## Background

The dairy sector is of the atmost importance in the developing world, where more than 80% of people live in rural areas (1–4). In Ethiopia, traditional methods account for 97% of dairying (5), using on-farm animal feed formulations, traditional feed and feed storage, and on-farm milking processes and materials (6), and there is also an excessive call for using unprocessed raw milk due to a lack of refrigeration centers on farms and in households and ambient temperatures, as spoiled and contaminated milk causes significant food wastage and food-borne disease (7–9).

Aflatoxins (AFs) and other derivatives present in milk and milk products are challenging health issues in food safety because they are unaffected by pasteurization and processing. This toxin induces cancer in experimental animals, and the relatively large consumption of milk by children makes this food contaminant a worldwide concern (10).

Animal feed is the beginning of the food safety chain in the dairying process (11). A wide variety of naturally occurring microbes contaminate and spoil animal feed on the farm and at the storage site (12).

Animals used for food trigger over five million illnesses, 46,000 hospitalizations, and 1,458 human fatalities annually due to contamination with numerous pathogenic microbes (11, 13). Dairy animals may become exposed to pathogenic microbes through the ingestion of contaminated feed, drinking contaminated water, udder infections, and directly from the environment. Such contamination may transmit disease-causing microbes or toxic metabolites to humans, leading to foodborne illness, cancer, and childhood stunting (14).

Animal feeds are contaminated with mycotoxins (aflatoxins), which are harmful by-products of the Aspergillus fungus species, and are particularly significant (15). Animal feed mycotoxin contamination could occur on the farm before harvest or during storage (16).

Animal feeds can become contaminated with microbes from industrial activity, fecal contamination, environmental pollution, inadequate temperatures, insect activity, and endogenous toxins during harvesting, storing, processing, mixing, or transportation (17). The US Food and Drug Administration (FDA) and the Federal Food, Drug, and Cosmetic Act of 1906 have recommended that animal feed provided to animals be appropriately made and labeled, in a way that poses no risk to human health (17). The US Department of Transportation (18) and Agriculture's Animal and Plant Health Inspection Service (19) have also advised that animal feed be safely transported to ensure healthy animals and improve productivity while also contributing to the nation's economy (20).

In Ethiopian farm forage and formulations of hay, cut and carry pasture, home-grown cereals, commercial concentrates (noug seed cake, maize, and concentrate mixture of wheat bran), and cultural home-brewed beer by-products (atella) are the most common groups of livestock feeds (21). In the locality, atella is usually stored in large quantities and used for a longer time, and in all parts of Ethiopia, especially in the study area, it is increasingly replacing commercial concentrates (22). Tella is one of the most commonly home-brewed traditional local beers and its by-product or residue (atella) is used in dairy cattle feed (21, 23).

Due to the low hygienic standards in the processing facility and the high chance of microorganisms from bioaerosols, floors, and contamination of the food equipment, Ethiopian dairy farm animal feed practices of handling, storing, and feeding are currently susceptible to contamination (24). Dairy farmers are forced to buy and store animal feed in hazardous conditions on a floor that has frequent exposure to the contaminated environment and human waste due to the scarcity and high cost of animal feed and the small number of food processing companies that produce feed by-products (24, 25).

Despite the livestock sector's contribution to the livelihood and economy of the Ethiopian population, having been identified as a new source of economic growth in the Second Growth and Transformation Plan (GTP) (26), livestock productivity remains very low due to the inefficiency of feed, genetic material, and veterinary services. The constraints related to feed shortage, in quantity and quality; environmental conditions; human waste contamination; and traditional feed storage practices have been critical problems in Ethiopian livestock production (27, 28).

With inadequate harvesting, storage, transportation, and processing methods, the generation of AFs in animal feed significantly increases, with toxicogenic, hepatogenic, carcinogenic, nephrotoxic, immunosuppressive, and mutagenic effects. Acute aflatoxicosis in humans is more likely to occur in developing nations due to factors like insufficient food supplies, a lack of infrastructure for AF management, and environmental factors that encourage AF growth (29).

Therefore, this study aims to evaluate animal feed AF contamination and its predictors among dairy farms in the South Gondar Zone (SGZ) of Ethiopia. This offers insight into how to lessen the risk of zoonosis, food hazards, and other public health issues, using a One Health strategy.

## Materials and methods

### Study area, study design, setting, and period

The study was conducted in the South Gondar zone of the Amhara National Regional State. The South Gondar zone is located 660 km northwest of Addis Ababa. The geographic location of the zone lies between 11° 02′-12° 33′ North latitude and 37° 25′-38° 43′ East longitude. The zone is characterized agro-ecologically as highland (Dega) and mid-altitude (Woina Dega), and the altitude ranges from 1,500 to 3,200 meters above sea level. The study area is generally characterized by its rugged topography from the place called Gunna Mountain (4,231 m) to the mid-altitude of the Dera district at the Lake Tana border. The annual minimum and maximum temperatures are ~17 and 27 degrees Celsius, respectively. Rainfall distribution is largely monomodal from June to mid-September. The heaviest rain usually occurs during July and August, and the mean annual rainfall varies widely from 500 to 1,600 mm.

A community-based experimental cross-sectional study was conducted from February 2021 to September 2021. In this study, 1,338 dairy farmers living in SGZ, Ethiopia, participated. The total sample size (100 dairy farmers) was proportionally allocated to each of the selected woredas using the total dairy farms of each selected woreda, total dairy farms of SGZ, and total sample size.

A systematic random sampling technique was used to enroll the dairy farms using the K^th^ interval (1,338/100 = within the 13 intervals). Of the 10 woredas of SGZ, Ethiopia (Debre Tabor Town, Dera, Ebenat, Farta, Fogera, Lay Gayint, Libo Kemekem, Esite, Simada, Tach Gayint,), three woredas (Libo Kemekem, Esite, Lay Gayint) were randomly selected using the lottery method, and the total numbers of dairy farms selected were 39, 32, and 29, respectively.

### Sampling and sample preparation

#### Sample size determination

Due to the high cost of microbiological analysis and economic constraints, to determine the sample size, 90% standard error, 0.83 probability of occurrence, design effect equals 1, and desired confidence of 0.1 were used. An average AF prevalence of 83% in feeds around Addis Ababa by Gizachew et al. (30) was used. The sample size was determined using the method described by Fisher et al. (31), as detailed below. *N* = z^2^pqD/d2 Where: *N* = Sample size to be calculated, *Z* = 1.96 at 90% standard error, *P* = probability of occurrence 0.83, *q* = 1-p, *D* = design effect equals 1 (around Addis Ababa City), d2 = desired confidence of 0.1, *N* = 1.962 × 0.83 × 0.17 × 1/0.12. Therefore, the total number of samples was 108. The final sample size (*n*_f_) of the study was 100.

#### Animal feed collection and sample preparation

Among the 100 dairy farms, samples of each ingredient (100 atella samples, 100 hay samples, 100 commercial concentrates samples, and 100 cut and carry pasture samples) were obtained and analyzed separately. One of the main aims of this study is to assess the *AF* contamination levels of the most commonly used animal feed types. Animal feed samples were collected from animal farms. In the locality, atella (local brewery end-product), hay, mixed commercial concentrates, and cut and carry pastures were the major feed types used. Samples were cut into three-inch pieces and quartered to reduce the sample size. The cut samples were placed in a plastic bag; the air was excluded and the bag was then tightly sealed and promptly shipped. Approximately 250 g representative feed samples, one feed sample from each dairy farm, were collected aseptically in a universal sample container: a strong molded glass bottle (3^1^/_4_ in height × 11/8 in diameter) of one oz. (28 ml) capacity with a flat base and wide mouth. It was sterilized with a loose cap, which was then tightened. Samples were transported to the laboratory in a cold box with ice packs immediately after collection for processing and analysis.

### Survey on knowledge and practice

The knowledge and practices of dairy farmers related to animal feed handling, aflatoxin animal feed contamination, and animal feed storage were assessed using a pretested structured questionnaire. Their knowledge was assessed using 14 pretested structured questionnaires, whereas the practices pretested structured questionnaire consisted of 9 of 100 samples adapted from previous studies (31). After the pretest, the knowledge and practices questionnaires were narrowed down to 9 and 6 pretested structured questionnaires, respectively. We employed the Crombath alpha to check the internal consistency, and the value was 0.8.

### Determination of *AFs* from animal feed

The quantitative measurement of the *AF* concentration of animal feed samples was determined by competitive Enzyme immunoassay, using the RIDASCREEN^®^ aflatoxin Total Art. No.: R4701 for *AF* determination in cereals and feed. An enzyme immunoassay kit was used, which cross-reacts between the different AFs that appear in the feed. Therefore, reference should be made to the total AFs (32).

### Data processing and statistical analysis

The data were entered into Epi-Data version 3.1 and then exported to SPSS (version 21.0) for analysis. *AF* levels in feed were categorized legally and highly based on the laboratory results by comparing the internationally accepted limit set by the EU (5 μg/kg). The Percentage, frequency, mean, and standard deviation were calculated. Binary logistic regression analysis was used to assess statistical associations between the predictors and the outcome variables. Therefore, variables with *P*-value < 0.25 in the bi-variable logistic regression analysis were included in the multivariable logistic regression analysis. *P*-values ≤ 0.05 were considered statistically significant, and an adjusted odds ratio (AOR) with 95% confidence intervals was used to examine associations between predictors and outcome variables.

### Data quality assurance

To validate the data, Triplicate laboratory analysis was performed and sample collection, handling, storage, and extraction were conducted based on scientific protocols. All feed sample analysis was done in accordance with scientific standard laboratory procedures. Sterilization and disinfection of the instruments were conducted in accordance with international standard procedures (33–35).

## Results

### Socio-demographic and household characteristics

The number of cattle owned by the households ranged between 1 and 16, with a mean (SD) of 6 (2) per household. On average, 26% of dairy farms used atella as the main feed for their animals and only 27% of dairy farms had training in animal feed handling (Table 1).

**Table 1:** Socio-demographic characteristics of dairy farms in the locality of SGZ, Ethiopia (*N* = 100).

**Variables**	**Characteristics**	**Value**
Age (in years)	Mean (SD)	37.22 (13.5)
	23–37	22
	38–57	33
	58–77	45
Gender of respondent	Men	79
	Women	21
Marital status of the respondent	Single	7
	Married	87
	Widowed/widower	6
Educational level	No formal education	23
	Can read and write	18
	Up to secondary school	41
	College/university	18
The average number of cattle/households	Mean (SD)	6 (2)
Total milk production in litter per day	Mean (SD)	5.61 (0.083)
Had training in dairy production	Yes	27
	No	73
Have a separate animal house	Yes	71
	No	29
Types of animal feed used	Hay	71
	Cu and carry pasture	28
	Atella	69
	Mixed Concentrates	67
	Other	9
Most-used animal feed (≥3 days/ week)	Hay	11
	Cut and carry pasture	27
	Atella	26
	Mixed Concentrates	36
The average area available for cutting and supplying pasture, as well as for grazing livestock in Hectares per cattle/household	Mean	1.21
Type of grazing system	Zero grazing	32
	Open grazing	16
	Mixed grazing	52
Have animal feed storage facilities	Yes	88
	No	12
Animal feed storage time	≤3 months	41
	3–6 months	33
	≥6 months	26

NA, not applicable; up to secondary school, primary and secondary school; SD, standard deviation.

### *AF* contamination levels of animal feed

Of the total 100 feed samples analyzed, 96% (96/100) were positive for *AFs*, ranging from LOD ( ≤ 1.75 μg/kg) to 306.9 μg/kg (Table 2). The European Union (EU) limit of 5 μg/kg was exceeded by 52% (52/100) of feeds. A total of 67 feed samples used concentrates with at least 22.8 ug/kg of AFs, which is higher than the legally allowable limit, but only 23 samples were reported to have an AF level lower than 5 ug/kg. Finally, the analysis of variance (ANOVA) showed that the mean *AF* contamination was significantly different across the four animal feed samples analyzed with a *P*-value of 0.001 (Table 2).

**Table 2:** Contamination level of AFs in different animal feed ingredients.

**AFs (μg/kg)**	**Animal feed ingredients**				
	**Hay**, ***N*** = **100**	**Cut and carry pasture**, ***N*** = **100**	**Concentrates**, ***N*** = **100**	**Atella**, ***N*** = **100**	**ANOVA**
Mean ± SD	19.00 ± 8.76	27.89 ± 8.90	88.00 ± 33.71	67.10 ± 21.00	0.001
Median	22.03	65.59	112.88	71.77	
Minimum	LOD	LOD	22.8	7.7	
Maximum	76.34	121	306.9	169	
≥5 μg/kg (number of samples)	7	11	23	11	

LOD, lowest detection limit; SD, Standard Deviation.

### Associated factors of AF contamination in animal feed

In the final model, logistic regression analysis showed that educational status; having no formal education and only being able to read and write were more likely to increase the AF contamination level of animal feed ingredients by 6 times and 5 times, respectively. While having no animal feed storage facilities, having longer feed storage durations (≥6 months), using mixed concentrates as the main source of animal feed, and having no training in dairy production were more likely to increase the AF contamination level of animal feed ingredients by 5 times, 5 times, 7 times, and 8 times, respectively (Table 3).

**Table 3:** Logistic regression analysis of factors associated with *AF* levels in animal feed ingredients ≥5 μg/kg among dairy farms in SGZ, Northwest Ethiopia, 2021 (*n* = 100).

**Factors**	**AFs**				***P*-value**
	≤ **5** μ**g/kg**	≥**5** μ**g/kg**	**COR (95%CI)**	**AOR (95%CI)**	
**Educational status**					
No formal education	6	17	1.8 (0.9, 3.4)	6.4 (3.4, 9.2)	**0.036** ^*^
Can read and write	7	11	2.4 (1.2, 4.5)	5.2 (1.9, 8.9)	**0.031** ^*^
Secondary school	23	18	1.8 (0.2, 16.6)	8.6 (0.2, 52.9)	0.779
Above college	12	6	1.0	0.1	
**Feed storage facility**					
Yes	47	41	1.0	1.0	
No	1	11	4.7 (1.7, 12.8)	5.5 (1.7, 8.1)	**0.001** ^*^
**Feed storage duration**					
≤ 3 months	32	9	1.0	1.0	
3–6 months	11	22	16.8 (11.5, 23.8)	7.5 (0.6, 10.6)	0.33
≥6 months	5	21	13.5 (1.5, 23.7)	5.8 (3.3, 8.2)	**0.011** ^*^
**Type of animal feed commonly used**					
Hay	18	7	1.0	1.0	
Cut and carry pasture	14	11	3.8 (0.4, 33.1)	8.7 (0.6, 13.76)	0.098
Atella	14	11	13.5 (1.5, 23.7)	3.8 (3.3, 4.2)	0.711
Mixed concentrates	2	23	7.8 (0.4, 33.1)	7.8 (0.6, 11.76)	**0.011** ^*^
**Had training in dairy production**					
Yes	17	10	1.0	1.0	
No	31	42	2.9 (2.4, 6.3)	8.2 (2.4, 15.6)	**0.03** ^*^
**Animal feed administrator knowledge of animal feed aflatoxin contamination**					
Yes	47	5	1.0	1.0	
No	7	41	5.56 (3.6, 6.2)	5.0 (4.2, 5.61)	**0.025** ^*^

* The association is significant at a P-value ≤ 0.05. A crude odds ratio (COR) is an odds ratio of one independent variable for predicting the dependent variable, whereas the adjusted odds ratio (AOR) holds other relevant variables constant and provides the odds ratio for the potential variable of interest, which is adjusted for the other independent variables included in the model.

## Discussion

Microorganisms found in animal feed can be transmitted to humans through the consumption of milk or milk products that are contaminated with animal feces and uncooked foods that have been contaminated by contact with uncooked milk products. Disease-causing infectious agents can be transmitted to animals through contaminated animal feed (17, 22).

Dairy farmers' knowledge of the processing of animal feed and the microbiological contamination of feed was found to be generally insufficient; only 22% were aware of this issue, and only 6% accurately described feed aflatoxin contamination. This result was low when compared to studies conducted in other parts of Ethiopia (36), Nairobi (37), and Kenya (38). The adoption of technology to prevent and control animal feed AF contamination may be severely impacted by a lack of understanding about the risks associated with animal feed, which could also result in improper handling of animal feed and harm both animal and human health (39).

In this study, of the 100 feed samples analyzed, 96 (96%) were contaminated with *AFs*, and the contamination level obtained in this study was comparable with many country-specific studies (22, 36) but higher than the findings of other studies conducted in Egypt (40) two studies in Turkey (41). The study also revealed that 52% of feeds exceeded the European Union (EU) limit of 5 μg/kg, which is comparable with a study conducted in Addis Ababa, higher than the results reported in Peru (42) and Argentina (43), and lower than the results of studies in Egypt (44) and Turkey (45). This variation could be due to feed originating from different agroecological conditions, under different feeding practices, different climates, and feed storage conditions.

In an analysis of associated factors, several factors may impact the occurrence and levels of *AFs* in animal feed. In this study, animal feed from dairy farmers who had no formal education and who could only read and write had a 6.4 and 5.2 times higher risk of *AF* contamination above the limit set by the EU than dairy farmers who had attended secondary and college education, respectively. The lack of sufficient knowledge, i.e., a poor understanding of the concept of AFs is common in many developing countries, which may facilitate high aflatoxin exposure through contaminated feeds, leading to human disease (46).

Dairy farmers with awareness of feed *AFs* may have information on the source and will protect feed and cows from *AF* exposure and, ultimately, safeguard feed from contamination. Furthermore, this result is supported by the fact that dairy farmers with no training in dairy production were 8.2 times more likely to experience AF contamination in animal feed above the limit set by the EU. AF intoxication leads to a decrease body weight and feed intake. The gross and microscopic changes in the aflatoxin groups were more pronounced with the increase in the level of the toxin present in the feed. Decreases in testicular size and volume and reproductive hormones like LH, prolactin, and testosterone were evident with increased levels of AFs in animal feed (47).

In comparison to those who had feed storage facilities, dairy farmers who had no animal feed storage facilities were shown to be 5 times more likely to have *AF* contamination above the limit set by the EU. High-humidity and high-temperature exposure, indoor storage in plastic bags, long-term storage, and the lack of raised ventilated platforms for storage may be conducive to the accumulation of molds and aflatoxins (13).

In this study, feed storage duration was also found to be a major factor in high AF contamination. Animal feed stored for a longer duration (≥6 months) was 5.8 times more likely to have AF contamination levels above the EU limit than animal feed stored for a shorter duration (≤ 6 months). This finding is supported by a study conducted in Kenya (46), which reasoned that the lengthy storage of animal feed due to a shortage of animal feed and lack of grazing fields and storing animal feed in restricted, closed rooms may favor and facilitate mold growth and contamination of the feed.

A Kenyan study revealed that concentrate feeds increase the likelihood of higher AF levels in comparison to other feeds, indicating that the transfer of veterinary and milk-borne pathogens from the environment of dairy farms to milk is a highly connected and complex process (the spread of AFs between humans and animals and their occurrence in the environment can result in milk becoming contaminated with pathogens). Despite its great importance, the dairy industry is challenged by many complicated technical and non-technical constraints. Feed shortage and microbial contamination due to easily preventable and avoidable factors are the key problems in livestock production, particularly among smallholder farmers (39). To prevent animal feed contamination, it is crucial to take into account a One-Health approach with relevant food safety and quality laws (48).

### Limitations of the study

Our study is one of the few in Ethiopia to provide information on the problem of animal feed microbial contamination. The limitations of this study include the small sample size, which was limited to 100 animal feed samples due to the high cost of reagents and supply, and the fact that AF levels in milk were not measured. Another limitation was that the study was conducted during the dry seasons due to resources and time constraints, and the contamination of AFs may vary throughout the seasons.

## Conclusion

The level of AF contamination in the most common feedstuffs, cow udders, and water used to wash milkers' hands and utensils used on dairy farms in the South Gondar zone of Ethiopia was relatively high. Low educational status, long feed storage durations, using mixed concentrates as the main source of animal feed, and having no training in dairy production were the significant factors associated with animal feed *AF* contamination levels.

Therefore, dairy farmers should be educated on the prevention mechanisms and physical detection methods of animal feed spoilage and on animal feed preparation and feeding. Ethiopia is known for different non-conventional animal feeds in different parts of the country, like atella in the study area, and efforts should be made to consider these as a major alternative, as cheap sources of feed. However, further investigation and research are needed to ascertain the nutritional value and improve the nutritive quality of feed in the interest of better livestock production. The One Health strategy should therefore receive special consideration.

## Data availability statement

The raw data supporting the conclusions of this article will be made available by the authors, without undue reservation.

## Ethics statement

Ethical approval was obtained from the Institutional Review Board, the Research Committee of Debre Tabor University, College of Health Sciences. The sampling of atella, hay, commercial concentrates, cut and carry pasture, swabs from cow udders, and water was carried out with the full consent of the head of the Farm.

## Author contributions

FT, ZM, and CY conducted the survey, conceived the design, interpreted the findings, and were responsible for the overall content as a guarantor. AAms, BD, AAmb, and HD planned the study, selected and assessed the quality of the study, extracted the data, interpreted the results, and reviewed the manuscript. All authors read and approved the manuscript.
